# Mother-to-child transmission of HIV: experience at a referral hospital in Saudi Arabia

**DOI:** 10.4103/0256-4947.59367

**Published:** 2010

**Authors:** Jameela Edathodu, Magid M. Halim, Muneera Bin Dahham, Abdulrahman A. Alrajhi

**Affiliations:** From the Section of Infectious Diseases, Department of Medicine, King Faisal Specialist Hospital and Research Centre, Riyadh, Saudi Arabia

## Abstract

**BACKGROUND AND OBJECTIVES:**

The rate of mother-to-child transmission of human immunodeficiency virus (HIV) type 1 has been reported to be high in Saudi Arabia. We report the rate of such transmission among a cohort of HIV-infected women enrolled in an HIV program at a tertiary care facility in Riyadh.

**METHODS:**

All HIV-infected women who became pregnant and delivered during their follow-up between January 1994 and June 2006 were included in this study. HIV viral load and CD4+ T-lymphocyte count near-term, the mode of delivery, and the HIV status of the newborn at 18 months were recorded. All women were counseled and managed according to the three-step PACTG 076 protocol.

**RESULTS:**

Of 68 HIV-infected women in the cohort, 31 had 40 pregnancies; one aborted at 13 weeks gestation. The mode of delivery was elective cesarean delivery in 28 pregnancies (70%) at 36 weeks gestation, and 11 (27.5%) had normal spontaneous vaginal delivery. The median CD4+ T-lymphocyte count near-term was 536 cells per cubic millimeter and the median viral load for 25 pregnancies was 1646 copies/mL, with only nine pregnancies (22.5%) having viral loads of more than 1000 copies/mL. Fourteen pregnancies (35%) had undetectable HIV prior to delivery. All patients were taking antiretroviral therapy during pregnancy and delivery. All 39 newborns tested negative for HIV infection at the age of 18 months; none of the newborns was breastfed.

**CONCLUSIONS:**

Contrary to previous local experience, diagnosis, management, and antiretroviral therapy almost eliminated mother-to-child transmission of HIV-1 in our patient population.

Mother-to-child transmission (MTCT) of the human immunodeficiency virus (HIV) was noted very early in the history of acquired immune deficiency syndrome (AIDS).^1^,^2^ As the epidemic of HIV/AIDS grew, women constituted 50% of infected people, and up to 60% in certain developing countries.^2^ Subsequently, MTCT now is the cause of almost all pediatric HIV infections.^2^ There is clinical and laboratory evidence to support several possible mechanisms for MTCT, including maternal disease state and viral load, fetal exposure to infected maternal body fluids during gestation and delivery, as well as breastfeeding.^3^–^8^ There is now sufficient evidence to suggest that transmission during late pregnancy and the intrapartum period contributes relatively more to the overall rate of vertical transmission than during the early intrauterine period.^6^ In the absence of any intervention, the rate of MTCT is reported to be 15% to 30 %.^8^ The Pediatric AIDS Clinical Trials Group (PACTG) Protocol 076 demonstrated that a three-part regimen that included zidovudine, another nucleoside reverse transcriptase inhibitor and a protease inhibitor could reduce the risk of MTCT of the HIV virus by nearly 70%.^7^,^9^

Data on the epidemiology of HIV-1 infection from the Middle East are limited. An 18-year surveillance of the epidemiology of HIV/AIDS in Saudi Arabia showed 1285 citizens to be infected with the HIV virus: 26.5% of them were women, mostly in the reproductive age group; 12.1% were children younger than 14 years old, and more than 53% of infected children had acquired the virus from their mothers.^10^ In another report from Riyadh, perinatal transmission accounted for 63% of all HIV infections in children.^11^ Reports on pregnancy outcome in women who are HIV-infected from the region and from Saudi Arabia are limited. We present the data on pregnancy outcome for HIV-infected women who delivered at our facility.

## METHODS

King Faisal Specialist Hospital and Research Centre (KFSHRC) is one of the referral centers for HIV-infected individuals in Saudi Arabia. The HIV Service of the Section of Infectious Diseases follows more than 500 HIV-infected adults. The screening test was done using AxSYM® HIV 1/2 gO MEIA (Abbott Laboratories, Abbott Park, IL, USA), is used to confirm the diagnosis of HIV infection. Abbot real-time PCR assay (Abbott RealTime M2000 rt, Abbott Laboratories, Abbott Park, Illinois, USA) is used used for the quantitation of HIV-1 virus. The lower limit of HIV viral load with this assay is 40 copies/mL. CD4+ count is done by standard flow cytometry using FACSCalibur™ (Becton Dickinson, San Jose, CA, USA).

Data were available for 102 HIV-infected women who were registered in the clinic. Sixty-eight were married, 13 were single, 17 were widowed, and 4 were divorced at the time of the study. Women older than 55 years of age, and pregnancies and deliveries that occurred before referral to our institution were excluded from the study. Data on demographics, number of pregnancies between January 1994 and June 2006, abortions, and mode of delivery were collected. Information was obtained about the use of zidovudine, viral load, and CD4+ T-lymphocyte count at about 36 weeks of gestation. Charts of the newborn were reviewed to collect data on the results of HIV antibody screening and HIV PCR at 18 months of age.

All women included in this study had been managed according to the three-step PACTG 076 protocol.^9^ The antenatal component consisted of 300 mg zidovudine orally twice daily, initiated between 14 and 34 weeks gestation (if not already on antiretroviral therapy) in addition to another nucleoside reverse transcriptase inhibitor (lamivudine in most cases) and a protease inhibitor, either nelfinavir or lopinavir/ritonavir, which were continued throughout the pregnancy. Women already on antiretroviral therapy for HIV infection were continued on their regimen, except that efavirenz was discontinued if part of the regimen. During labor, intravenous zidovudine was given as a loading dose of 2 mg/kg over one hour, followed by a continuous infusion of 1 mg/kg per hour until the cord was clamped. The postpartum component consisted of the administration of zidovudine syrup (2 mg/kg orally four times daily) to the infant for the first six weeks of life, beginning 8 to 12 hours after birth for the prevention of MTCT. Elective cesarean delivery was recommended until 2003 after which, vaginal delivery became the standard in mothers with viral load below 1000 copies/mL, unless the patient opted for cesarean delivery.

## RESULTS

Thirty-one women with 40 pregnancies were identified as fitting the inclusion criteria for the study. One pregnancy aborted at 13 weeks gestation, the patient had a viral load of 3607 at that time and she was treatment-naïve. The reason for the abortion was unclear. The mode of delivery was elective cesarean delivery in 28 pregnancies (70%) at 36 weeks gestation; 11 (27.5%) had normal spontaneous vaginal delivery. The median CD4+ T-lymphocyte count at about the time of delivery was 536 cells/mm^3^ (mean 574, range 183-1142 cells/mm^3^), and the median viral load at about delivery time for 25 pregnancies was 1646 copies/mL (73-59071), with only 9 (22.5%) pregnancies having viral loads of more than 1000 copies/mL. Fourteen pregnancies (35%) were receiving highly active antiretroviral therapy (HAART), had undetectable HIV prior to delivery. All were on antiretroviral therapy during pregnancy and delivery. All the newborns were tested at the end of 18 months and tested negative for the HIV-1 screening and PCR assay. Breast feeding was strictly prohibited and complied with by the patients in the study.

## DISCUSSION

In a study looking at horizontal versus vertical transmission in 19 HIV-infected people and their contacts, Al Nozha et al found that all nine children born to HIV-infected mothers were HIV-infected.^12^ None of the mothers were aware of their serostatus, and none had received antiretroviral therapy, or avoided breast feeding. Our data in this study demonstrate reassuringly successful prevention methods for MTCT. While looking at the reasons for HIV testing in Saudi Arabia, Alrajhi et al^13^ showed that the majority of the women with HIV infection acquired the infection from their husbands. Both the natural instincts for motherhood and social pressures culminated in pregnancies in our Saudi population of HIV-infected women. Counseling for conception and childbirth is an essential component of healthcare provided to HIV-infected women. The majority of the pregnancies in our report were planned and coordinated with care providers to ensure tolerance, adherence, and response to antiretroviral therapy before conception. In our hospital, we follow the Public Health Service Task Force recommendations for the management of pregnant HIV-infected women.^14^ Our comprehensive care team for HIV-infected women includes social service workers, HIV counselors, pharmacists, and obstetricians.

The MTCT rate of 0% in our study is probably related to the small number of patients. However, other reports like the AmRo study from Netherlands,^15^ also had an MTCT rate of 0% among 143 pregnancies. Use of HAART has been associated with preterm deliveries, preeclampsia, and low birth weight.^15^ In our study, we had no cases of preeclampsia or preterm delivery; all cesarean deliveries were done electively around 36 weeks of gestation.

MTCT of HIV-1 is multifactorial and may occur even in women with undetectable viral loads.^16^ The risk of vertical transmission can be decreased remarkably with elective cesarean section and zidovudine prophylaxis. The PACTG 076 Trial showed clearly that vertical transmission of HIV-1 could be reduced considerably, irrespective of the maternal viral load, with the use of zidovudine.^7^,^9^ The effectiveness of zidovudine in reducing MTCT has been shown to be consistent. Breastfeeding increases the transmission of HIV-1 to the infant by 5-20%. A study from Saudi Arabia looking at the mode of transmission of HIV-1 infection in children showed that 63.5% acquired the infection vertically; 93% were breast fed for almost two years.^11^ The compliance to HAART therapy and abstinence from breastfeeding^17^ could have contributed to the good outcome in our cohort.

In conclusion, contrary to previous local experience with MTCT, we believe that diagnosis, counseling, antiretroviral therapy, elective cesarean delivery, early antiretroviral therapy for the newborn, and avoiding breastfeeding have contributed to eliminating MTCT among our HIV-infected, pregnant women.
